# Safety and Clinical Outcomes of Transvenous Lead Extraction for Cardiac Device Infections in the Very Elderly

**DOI:** 10.1002/joa3.70245

**Published:** 2025-12-06

**Authors:** Khalid Sawalha, John P. Marenco, Laurence M. Epstein, Shayal Pundlik, Kyle Gobeil, Marshal Fox, Fadi Chalhoub

**Affiliations:** ^1^ Department of Cardiology UMass Chan Medical School—Baystate Springfield Massachusetts USA; ^2^ Department of Cardiology, Zucker School of Medicine at Hofstra/Northwell North Shore University Hospital Manhasset New York USA

**Keywords:** device infection, elderly, transvenous lead extraction

## Abstract

**Background:**

The increasing use of cardiac implantable electronic devices (CIEDs) has led to a rise in transvenous lead extractions (TLE), particularly for device‐related infections. The elderly represent a growing subgroup undergoing TLE, but data on their outcomes are limited.

**Objectives:**

To evaluate the safety and in‐hospital outcomes of TLE in patients aged ≥ 80 years with device‐related infections.

**Methods:**

We analyzed the National Inpatient Sample (NIS) from 2016 to 2020 to identify hospitalizations involving TLE for device‐related infections. Patients were stratified by age: < 80 years and ≥ 80 years. The primary outcome was in‐hospital mortality. Secondary outcomes included major procedural complications and length of stay. Multivariate logistic regression identified independent predictors of in‐hospital mortality and complications.

**Results:**

Among 30 670 patients who underwent TLE, 6530 (21.3%) were aged ≥ 80 years. In‐hospital mortality did not differ significantly between groups (4.0% vs. 4.6%, *p* = 0.40), nor did overall complication rates (6.7% vs. 6.9%, *p* = 0.81). However, elderly patients had higher rates of post‐procedural stroke (0.3% vs. 0.02%, *p* = 0.002) and bleeding (1.6% vs. 0.8%, *p* = 0.04). Independent predictors of mortality included chronic kidney disease (aOR 2.2, 95% CI: 1.2–4.2), cirrhosis (aOR 12.2, 95% CI: 1.1–133), and respiratory failure (aOR 50.7, 95% CI: 6–425). Elderly patients were more frequently discharged to rehabilitation facilities (40.3% vs. 25.5%, *p* < 0.001).

**Conclusion:**

Elderly patients undergoing TLE for infections had similar in‐hospital mortality and complication rates compared to younger patients. Age alone should not preclude TLE. However, increased risks of stroke and bleeding warrant targeted perioperative assessment. Further studies are needed to assess long‐term outcomes in this population.

## Graphical abstract

In this nationwide study of patients undergoing transvenous lead extraction for device‐related infections, very elderly individuals (≥ 80 years) had comparable in‐hospital mortality and complication rates to younger patients. These findings demonstrate that advanced age alone should not preclude extraction, though tailored risk assessment remains essential in this population.

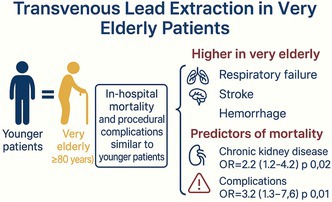

## 1 Introduction

The use of cardiac implantable electronic devices (CIEDs), including pacemakers and implantable cardioverter‐defibrillators (ICDs), has expanded substantially over the past few decades, driven by broadening clinical indications and improvements in device technology. As a result, device implantation has become increasingly common among elderly and medically complex patients. With the growing prevalence of CIEDs, there has been a corresponding rise in device‐related complications—particularly infections, which remain the leading indication for transvenous lead extraction (TLE) [1].

Despite advances in extraction tools and operator experience, there is a misconception that TLE is a high‐risk procedure particularly in older adults. This perception has contributed to hesitancy in referring elderly patients for TLE, even in the presence of class I indications such as device‐related infections. However, multiple studies have demonstrated that TLE can be performed safely in elderly populations, with low rates of procedural mortality and major complications [1, 2, 3].

Patients aged 80 years and older represent a growing subset of the CIED population requiring extraction. These individuals often present with increased frailty, a higher burden of comorbidities, and more complex extraction profiles, all of which may influence procedural outcomes. Although prior studies have reported favorable safety outcomes of TLE in octogenarians, these investigations are often limited by small sample sizes, single‐center designs, and high‐volume operators. As such, their findings may not be generalizable to broader, real‐world practice settings.

In this study, we used a nationally representative dataset to evaluate the safety and in‐hospital outcomes of TLE in patients aged 80 years and older. Our objective was to assess procedural complications, mortality rates, and predictors of adverse events in this increasingly relevant population using real‐world data reflective of diverse practice settings.

## 2 Methods

This study utilized data from the National Inpatient Sample (NIS) database for the years 2016–2020. The NIS is the largest publicly available all‐payer inpatient healthcare database in the United States and is maintained by the Agency for Healthcare Research and Quality (AHRQ). It includes data on approximately 8 million hospital stays annually, selected using a complex stratified probability sampling design. When weighted, the NIS represents more than 35 million hospitalizations from non‐federal, short‐term, general, and specialty hospitals nationwide, approximating 20% of all U.S. community hospitals. The database includes comprehensive information on patient demographics (age, sex, race, insurance status), primary and secondary diagnoses and procedures, hospitalization outcomes, resource utilization (e.g., total cost and length of stay), and discharge disposition. Strict safeguards are in place to protect the privacy of patients, providers, and institutions. The validity and reliability of the NIS have been well established, with its results shown to correlate closely with other national hospitalization discharge datasets. Institutional Review Board approval was exempted from full review for the current study because all data collection was derived from a de‐identified administrative database.

### 2.1 Study Population

We analyzed the NIS database to identify patients who underwent transvenous lead extraction using specific ICD‐10‐PCS codes: 02PA3MZ, 02PA4MZ, and 02PAXMZ. To ensure the accuracy and validity of our findings, we carefully selected codes specific to TLE and excluded those that may reflect lead revision or other procedures. Lead extractions performed due to device‐related infections (DRI) were identified by the presence of a diagnosis code indicating device infection in conjunction with a TLE procedure during the same hospitalization. Additionally, we captured cases where the primary admission diagnosis was sepsis, bacteremia, septic shock, or endocarditis, provided the patient underwent TLE during that same admission. Finally, patients were then categorized into two age groups: those younger than 80 years and those aged 80 years or older.

### 2.2 Study Variables and Outcomes

Comorbid conditions were identified using data from the Agency for Healthcare Research and Quality. Baseline patient characteristics were extracted from the NIS dataset, including age, sex, race/ethnicity, hospital size, and hospital teaching status (teaching vs. non‐teaching). Relevant comorbidities potentially impacting the primary outcome were identified using ICD‐10‐CM codes. These included congestive heart failure (CHF), coronary artery disease (CAD), chronic obstructive pulmonary disease (COPD), smoking, hyperlipidemia, peripheral vascular disease (PVD), hypertension, chronic kidney disease (CKD), among others listed in Table 1. Additional comorbidities were also identified to calculate the Charlson Comorbidity Index (CCI), a validated tool used to predict one‐year mortality based on comorbidities, with higher scores indicating more severe disease [4].

**TABLE 1:** Baseline characteristics of patients undergoing TLE for DRI dichotomized by age.

Variable %	Age < 80	Age ≥ 80	*p*
Number of patients	24 140	6530	—
Age (mean + SD)	62.9 ± 0.22	84.6 ± 0.1	—
Female	29.8	31.8	0.2
White	72.3	84.1	< 0.001
Black	15.2	4.7	
Hispanic	8.1	6.8	
Teaching hospital	88.5	85.9	0.04
Hospital size			0.08
Small size	7.4	9	
Medium size	19.2	20.8	
Large size	73.4	70.2	
Coronary artery disease	46.1	54.9	< 0.001
Congestive heart failure	66.2	59.6	< 0.001
Chronic kidney disease	36.6	42.7	< 0.001
Diabetes	45	31.7	< 0.001
Peripheral vascular disease	5.7	6.7	0.29
Chronic obstructive pulmonary disease	17.5	16.3	0.4
Hypertension	22.3	26.8	0.003
Coronary artery bypass graft	13.4	21.1	< 0.001
Liver cirrhosis	0.9	0.3	0.02
Anemia	16.2	17.5	0.28
Smoking	24.8	27.9	0.05
Morbid obesity	8.9	1.9	< 0.001
Dialysis	5.9	3.8	0.01
CCI = 1	14.4	17.5	< 0.001
CCI = 2	18.9	15.9	
CCI ≥ 3	59.2	55.7	
Obstructive sleep apnea	16.5	10.3	< 0.001
Obesity, BMI > 30	11	5.8	< 0.001
Underweight, BMI < 18	1.7	2.9	0.01

The primary outcome of the study was in‐hospital mortality. Secondary outcomes included the length of hospital stay and periprocedural complications associated with TLE. We focused on complications commonly reported in the literature, including pericardial events (tamponade, hemopericardium, and pericardial effusion), pulmonary complications (pneumothorax, post‐procedure respiratory failure, and hemothorax), and life‐threatening hemorrhage requiring transfusion, along with other complications detailed in Table 2. To improve specificity, we excluded codes that may represent chronic comorbidities rather than acute procedural complications. Procedural complications were identified using ICD‐10‐CM codes specifying post‐procedural adverse events (e.g., post‐procedural stroke, hemorrhage, respiratory failure). These codes are assigned when a complication occurs after the completion of a surgical or procedural intervention during the same hospitalization. The NIS does not differentiate between intraoperative and postoperative timing; therefore, complications recorded here represent all adverse events temporally associated with the procedure but not distinguishable by exact timing. Additional outcomes included the identification of independent predictors of in‐hospital mortality and procedural complications. We also examined temporal trends in TLE utilization over time among patients aged 80 years and older.

**TABLE 2:** Mortality, length of stay, and complications of patients undergoing TLE for DRI stratified by age.

	Age < 80	Age ≥ 80	*p*
Mortality (%)	4.6	4.0	0.4
Mean LOS (days)	13.7	11.4	0.08
Rehab transfer (%)	25.5	40.3	< 0.001
Procedural complications ≥ 1%	6.9	6.7	0.81
Pericardial effusion (%)	3.4	2.8	0.37
Tamponade (%)	0.9	0.7	0.6
Respiratory failure (%)	0.9	0.2	0.01
Pneumothorax %	0.4	0.8	0.2
Post‐procedure stroke (%)	0.02	0.3	0.002
Post‐procedure hemorrhage (%)	0.8	1.6	0.04
Post Procedure cardiac arrest %	0.08	0.3	0.2
Post Hemothorax %	0.5	0.3	0.5

Abbreviation: LOS, length of stay.

### 2.3 Statistical Analysis

Continuous variables were presented as weighted means ± standard deviations, while categorical variables were summarized as frequencies and percentages. Independent *t*‐tests were used to compare continuous variables, and Chi‐square tests were applied to compare categorical variables. All analyses incorporated discharge‐level weights to produce nationally representative estimates of hospitalized patients in the United States.

Univariate and multivariate logistic regression analyses were performed to assess associations between transvenous lead extraction and both primary and secondary outcomes. The multivariate models were adjusted for demographic variables, the Charlson Comorbidity Index (CCI), relevant clinical comorbidities, and hospital‐level characteristics. Length of stay (LOS) was analyzed using linear regression models, with log transformation applied to address the positive skewness of the LOS distribution.

Additionally, multivariable logistic regression was used to identify independent predictors of in‐hospital mortality and major procedural complications among patients undergoing TLE. Variables with a *p*‐value < 0.2 in univariate analysis were included in the multivariate regression model. A two‐tailed *p*‐value < 0.05 was considered statistically significant. All analyses were conducted using Stata version 15 (StataCorp LLC, College Station, TX, USA).

## 3 Results

**FIGURE 1:**
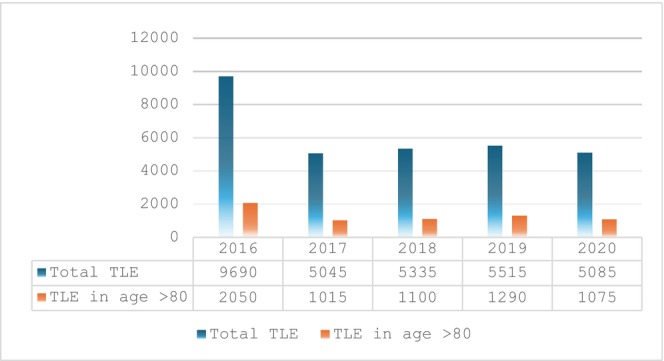
Transvenous Lead extraction for DRI from 2016 to 2020.

Over the five‐year period from 2016 to 2020, a total of 30 670 patients who underwent transvenous lead extraction (TLE) were analyzed (Table 1). Of these, 24 140 patients were under the age of 80, with a mean age of 62.9 years, and 6530 patients (21.3%) were aged 80 or older, with a mean age of 84.6 years. As shown in Figure 1, the annual number of TLE procedures decreased by approximately 40% after 2016. Among the very elderly cohort, 85.9% were White and the majority of patients, 78.2% were male. In contrast, the younger group included a higher proportion of Black patients (15.2%) compared to the elderly group (4.7%).

There were no significant differences between age groups regarding hospital size or teaching status. The most prevalent comorbidities among elderly patients were chronic kidney disease (42.7%), congestive heart failure (59.6%), and prior coronary artery bypass grafting (21.1%)., A higher Charlson Comorbidity Index (CCI) score was observed in the younger group, with 59.2% having a CCI > 3 compared to 55.7% in the elderly group, and 18.9% versus 15.9% for CCI > 2, respectively.

In the pooled patient sample, the overall all‐cause in‐hospital mortality rate was 4.3%. There was no statistically significant difference in mortality between younger and older patients (4.6% vs. 4.0%, *p* = 0.40). The average length of hospital stay was 13.7 days in the younger group compared to 11.4 days in the elderly group. A significantly higher proportion of elderly patients were discharged to rehabilitation facilities (40.3% vs. 25.5%, *p* < 0.001).

Overall, complication rates did not differ significantly between the two age groups. However, certain complications were more frequent in the elderly. Post‐procedural stroke occurred more often in the elderly group (0.3% vs. 0.02%, *p* = 0.002), as did post‐procedural bleeding (1.6% vs. 0.8%, *p* = 0.04) (Table 2). Among elderly patients who died during hospitalization, 10.3% experienced procedure‐related complications. Complications were more frequent in teaching hospitals (7.1%) compared to non‐teaching hospitals (4.4%), and higher in small hospitals (10.2%) than in medium (4.4%) or large hospitals (6.7%). However, these differences did not reach statistical significance. Patients with cirrhosis had a higher likelihood of complications (OR 6.2, 95% CI 0.5–70), and complication rates were also elevated among those with morbid obesity (16% vs. 6.4%), though neither association was statistically significant.

The results of the multivariate logistic regression analysis are summarized in Table 3. Significant predictors of in‐hospital mortality included chronic kidney disease (aOR: 2.2; 95% CI: 1.2–4.2; *p* = 0.02) and liver cirrhosis (aOR: 12.2; 95% CI: 1.1–133; *p* = 0.04). Procedural complications overall were independently associated with increased mortality (aOR: 3.2; 95% CI: 1.3–7.6; *p* = 0.01). Among specific complications, pulmonary complications significantly increased the risk of death (aOR: 8.6; 95% CI: 2.1–35.2; *p* = 0.003). Notably, respiratory failure emerged as the strongest predictor of mortality, with an adjusted odds ratio of 50.7 (95% CI: 6–425; *p* < 0.001).

**TABLE 3:** Independent predictors of mortality following TLE in patients ≥ 80 years.

	Odds ratio	Confidence interval	*p*
Female	1.6	0.84–3.1	0.15
Coronary artery bypass graft	0.48	0.2–1.2	0.14
Chronic kidney disease	2.2	1.2–4.2	0.02
Dialysis	2.6	0.8–8	0.1
Congestive heart failure	1.7	0.8–3.4	0.1
Cirrhosis	12.2	1.1–133	0.04
Underweight	2.1	0.5–9.9	0.3
Complications ≥ 1	3.2	1.3–7.6	0.01
Pericardial effusion	3	0.8–11	0.09
Respiratory failure	50.7	6–425	< 0.001
Pos hemothorax	46.1	1.7–710	0.02
Pulmonary complications	8.6	2.1–35.2	0.003

## 4 Discussion

In this nationally representative analysis of 30 670 patients undergoing TLE for device‐related infections, approximately one in five patients was aged 80 years or older. Despite concerns regarding procedural risk in this older population, we found no significant differences in all‐cause in‐hospital mortality (4.0% vs. 4.6%, *p* = 0.40) or overall procedural complication rates (6.7% vs. 6.9%, *p* = 0.81) between octogenarians and younger patients. Post‐procedural respiratory failure and pulmonary complications were more frequently observed in younger patients probably because of the higher comorbidities. Although rare among the elderly (0.2%), respiratory failure emerged as the strongest independent predictor of in‐hospital mortality (OR 50.7, 95% CI 6–425, *p* < 0.001). The very elderly had a higher incidence of post‐procedural stroke compared to the younger cohort (0.3% vs. 0.02%, *p* = 0.002). In the very elderly cohort, the presence of chronic kidney disease (OR 2.2, 95% CI 1.2–4.2, *p* = 0.01), cirrhosis (OR 12.2, 95% CI 1.1–133, *p* = 0.04), and post‐procedural complications (OR 3.2, 95% CI 1.3–7.6, *p* = 0.01) were all significantly associated with increased in‐hospital mortality.

There remains ambiguity in the literature regarding mortality after TLE in elderly patients. Some suggest that advanced age is associated with higher rates of complications and mortality [3, 5]. In Lin, A et al. meta‐analysis, the reported mortality rate among elderly patients was only 0.4% which is markedly lower than in our study, likely due to selection bias and the limited representation of very elderly individuals in the included studies [6]. In contrast, Bongiorni et al., utilizing data from the European Lead Extraction ConTRolled (ELECTRa) registry involving 73 centers across Europe, reported age > 68 years as a significant predictor of increased all‐cause mortality during hospitalization [7]. Similarly, a prior NIS‐based analysis from 2005 to 2012 of TLE procedures irrespective of indication showed no significant difference in mortality or complication rate among age groups regardless of device infection status [8]. Also, Husseini et al. reported mortality of 4.1% in elderly patients from 2003 to 2015 which is consistent with our study [9]. The variability in reported mortality after TLE in elderly patients likely reflects differences in study populations, definitions of “elderly,” and procedural indications, with some analyses underrepresenting very elderly or high‐risk individuals. Additionally, reliance on selective or limited single‐center datasets may underestimate the true risk of the procedure.

Certain complications specifically post‐procedural stroke and bleeding were more common in the elderly cohort, consistent with age‐related vulnerability to cerebrovascular and vascular injury, which highlights the importance of perioperative monitoring and individualized risk stratification. Post‐procedural respiratory failure was a strong predictor of in‐hospital mortality, aligning with prior studies that emphasize the serious impact of pulmonary complications in frail populations. Elderly patients are particularly vulnerable to respiratory failure after TLE due to reduced pulmonary reserve, diminished respiratory muscle strength, and a higher prevalence of pulmonary disease all of which impair their ability to tolerate procedural stress and recover from respiratory illness difficult [10]. Also, TLE is often a complex and prolonged procedure typically performed under general anesthesia, which introduces additional risk in older adults.

Our analysis also revealed that comorbidities such as chronic kidney disease (aOR 2.2) and cirrhosis (aOR 12.2) were independently associated with in‐hospital mortality. The Charlson Comorbidity Index was slightly higher in younger patients, which may reflect either more careful case selection among elderly individuals or undercoding in elderly individuals. Elderly patients with a BMI < 18 had higher odds of mortality (OR 2.1), though this was not statistically significant. Prior CABG appeared to be protective (OR 0.48), but did not reach significance. While previous cardiac surgery has been suggested as protective against perforation due to adhesions that may limit bleeding, it does not reduce mortality. In fact, if perforation occurs in this setting, outcomes may be worse due to the technical challenges of managing such injuries during redo sternotomy [11, 12]. While complication rates were similar between teaching and non‐teaching hospitals, complications were numerically higher in smaller hospitals, suggesting that institutional volume may influence outcomes [13]. Additionally, teaching hospitals had a slightly higher rate of complications, which may be attributable to the referral of more complex cases and the performance of higher‐risk procedures in these centers. The trend of TLE in elderly patients showed that the highest number of procedures occurred in 2016, followed by a 40% decline in 2017, after which the numbers plateaued.

Notably, despite shorter average lengths of stay, the elderly were more likely to be discharged to rehabilitation facilities (40.3% vs. 25.5%), indicating higher post‐discharge dependency, which is expected given the greater baseline frailty in this group that also might be contributing to mortality in this age group as shown in previous studies [14]. These data emphasize that in elderly patients, the success of TLE should not be evaluated solely in terms of in‐hospital mortality or procedural complications, but also in terms of long‐term recovery, functional independence, and quality of life.

There are several notable strengths to our study. First, it utilizes a large, nationally representative dataset, enabling a comprehensive assessment of TLE outcomes across a wide range of hospitals and patient populations. We emphasize that the NIS encompasses hospitals of all sizes and teaching statuses across the United States, capturing cases performed by operators with varying experience levels. This broad representation allows our analysis to reflect real‐world national practice patterns. In contrast to prior studies—many of which are from single high‐volume centers and include only small samples of elderly patients—our analysis captures real‐world practice across institutions of varying sizes and procedural volumes. This enhances the generalizability of our findings and provides a more accurate and applicable estimate of procedural risk in elderly patients. Moreover, we believe that in many hospitals, TLE is performed by a single or very few operators, meaning that data from single‐center studies often reflect the outcomes of individual operators rather than broader institutional practice. This introduces selection and operator bias into the reported data. For example, a study conducted at a high‐volume center in Italy reported zero mortality among 1316 patients undergoing TLE, despite 81% of cases being infection‐related [15]. This strikingly low mortality highlights the potential for bias in highly specialized centers and underscores the importance of assessing outcomes in more representative settings.

On the other hand, several limitations of our analysis should be considered when interpreting the findings. These primarily relate to the retrospective nature of the study and reliance on an administrative database, which uses ICD‐10 codes to identify procedures, complications, and comorbidities. While TLE is a major inpatient procedure and thus unlikely to be miscoded, comorbidity coding may be less accurate, potentially underestimating patients' true risk profiles. Additionally, the NIS lacks key clinical details—such as lead type, extraction technique, dwell time, and operator experience which are known to influence procedural outcomes.

Our analysis is limited to in‐hospital events; thus, we were unable to assess long‐term mortality, device reimplantation, or post‐discharge recovery and quality of life. Although we adjusted for comorbidity burden using the Charlson Comorbidity Index, the dataset does not include geriatric‐specific variables such as frailty, cognitive impairment, or functional status, which are important predictors of outcomes in elderly patients. Furthermore, while hospital size and teaching status were included, other institutional characteristics such as procedural volume and the presence of dedicated extraction teams were not available.

Importantly, given that infectious indications carry higher risk, a greater proportion of elderly patients undergoing extraction for infection could partially explain the observed differences in mortality. Because the NIS database relies on administrative coding, it was not possible to distinguish between localized pocket infections and systemic infections such as lead‐associated endocarditis or sepsis. This distinction is clinically important because systemic infections are associated with higher morbidity, more urgent surgical intervention, and worse hemodynamic stability compared with isolated pocket infections. Therefore, our findings reflect aggregate outcomes for all device‐related infections rather than specific subtypes. Additionally, several procedural factors known to influence TLE outcomes could not be assessed using the NIS dataset. These include the type of device (pacemaker vs. ICD), the presence of coil leads such as SVC coils, lead dwell time, and the use of specific extraction techniques or tools (laser versus mechanical extraction sheaths). Longer dwell times and the need to extract shock leads are associated with increased procedural complexity, longer fluoroscopy times, and higher complication rates. The absence of these variables in the dataset may have led to residual confounding and limits our ability to perform a more granular risk stratification. These limitations underscore the need for prospective studies with more granular clinical data to better inform decision‐making in this growing patient population.

## 5 Conclusion

In this national analysis of transvenous lead extraction for device infections, very elderly patients had comparable in‐hospital mortality and complication rates to younger patients, indicating that age alone should not preclude TLE. Nonetheless, higher rates of comorbidities and specific complications such as stroke and bleeding in the elderly underscore the importance of individualized perioperative risk evaluation.

## Conflicts of Interest

The authors declare no conflicts of interest.
